# Metabolic regulation by biomaterials in osteoblast

**DOI:** 10.3389/fbioe.2023.1184463

**Published:** 2023-05-30

**Authors:** Zhengyang Kang, Bin Wu, Luhui Zhang, Xinzhi Liang, Dong Guo, Shuai Yuan, Denghui Xie

**Affiliations:** ^1^ Department of Orthopedics, The Second People’s Hospital of Panyu Guangzhou, Guangzhou, China; ^2^ Department of Joint Surgery and Sports Medicine, Center for Orthopedic Surgery, Orthopedic Hospital of Guangdong Province, The Third Affiliated Hospital of Southern Medical University, Guangzhou, China; ^3^ Guangdong Provincial Key Laboratory of Bone and Joint Degeneration Diseases, The Third Affiliated Hospital of Southern Medical University, Guangzhou, China; ^4^ Guangxi Key Laboratory of Bone and Joint Degeneration Diseases, Youjiang Medical University For Nationalities, Baise, China

**Keywords:** advanced materials, bone regeneration, metabolic regulation, osteoblast, α-Ketoglutaric acid based polymer

## Abstract

The repair of bone defects resulting from high-energy trauma, infection, or pathological fracture remains a challenge in the field of medicine. The development of biomaterials involved in the metabolic regulation provides a promising solution to this problem and has emerged as a prominent research area in regenerative engineering. While recent research on cell metabolism has advanced our knowledge of metabolic regulation in bone regeneration, the extent to which materials affect intracellular metabolic remains unclear. This review provides a detailed discussion of the mechanisms of bone regeneration, an overview of metabolic regulation in bone regeneration in osteoblasts and biomaterials involved in the metabolic regulation for bone regeneration. Furthermore, it introduces how materials, such as promoting favorable physicochemical characteristics (e.g., bioactivity, appropriate porosity, and superior mechanical properties), incorporating external stimuli (e.g., photothermal, electrical, and magnetic stimulation), and delivering metabolic regulators (e.g., metal ions, bioactive molecules like drugs and peptides, and regulatory metabolites such as alpha ketoglutarate), can affect cell metabolism and lead to changes of cell state. Considering the growing interests in cell metabolic regulation, advanced materials have the potential to help a larger population in overcoming bone defects.

## 1 Introduction

Bone defects are a prevalent form of clinical injury, and the bone possesses some inherent regenerative capacity. Generally, small bone defects such as fractures do not require surgical intervention; however, larger bone defects exceeding a critical size are challenging to heal spontaneously and require medical intervention (Koons et al., 2020). Bone defects is originated from a range of causes, including trauma, degenerative disease, congenital defects, and surgical resection of tumors (Leach and Whitehead, 2017). Hence, the importance of clinical intervention increases significantly (Roddy et al., 2018). The primary method for treating bone defects is through bone grafting. There are currently three primary types of bone defect repair materials: autologous bone, allogeneic bone, and artificial bone regeneration materials. Autologous bone grafts are considered as the “gold standard” for bone regeneration (C. Wang et al., 2022), but the supply of autologous bone is limited and the donor site is susceptible to complications related to healing. Allogeneic bone grafts can fill large bone defects, but the risks of fibrous bone discontinuity, inflammation, and microfracture expansion can lead to post-implant failure (Qu et al., 2021). Considering the issues associated with autologous and allogeneic bone grafts, as well as the high clinical demand for bone grafts, it is anticipated that artificial bone regeneration materials, especially the biomaterials, will become a critical avenue for bone repair.

The growing demand for synthetic materials that facilitate bone regeneration in the field of regenerative medicine underscores the necessity for more expansive investigations aimed at establishing a thorough comprehension of the fundamental mechanisms that regulate cellular reactions to biomaterials (Murphy et al., 2014). Cell metabolism is the set of chemical reactions that occur in living organisms to maintain life. These reactions are essential for energy production, growth, repair, and other cellular functions. Cell metabolism can be divided into two main categories: catabolism and anabolism. Catabolism is the breakdown of larger molecules into smaller ones, releasing energy in the process. For example, the breakdown of glucose in the presence of oxygen, known as cellular respiration, produces energy in the form of ATP and releases carbon dioxide and water as waste products. Anabolism is the opposite of catabolism and involves the synthesis of larger molecules from smaller ones, requiring energy input. For example, the synthesis of collagen which is the most abundant protein in bone tissue and is responsible for providing structural support and strength, requires energy input. Metabolism is regulated by a complex network of enzymes, hormones, and other signaling molecules that respond to changes in the internal and external environment of the cell (Newgard, 2017). Despite recent advances in understanding how materials influence cellular metabolism, there is a growing need for further investigation into the complex interactions between cells and their surrounding microenvironment, including the physical and chemical properties of the materials in which they are embedded, as well as the signaling pathways and biochemical reactions that are triggered by these materials. The interplay between a cell and its microenvironment remains incomplete. The inadequacy of our current knowledge regarding the influence of material cues on intracellular metabolic pathways is particularly conspicuous. This is especially significant considering that cell metabolism is presently recognized as a cascade of intracellular occurrences that interact dynamically with signaling and gene expression, ultimately influencing cellular decision-making. (Ji et al., 2020; Shyh-Chang, Daley, and Cantley, 2013; Jan A van der Knaap and Verrijzer 2016b). In this review we provide a detailed discussion of the mechanisms of bone regeneration and an overview of the regulation of cell metabolism. Furthermore, this article explores the potential impact of bone defect repair materials on cell metabolism and behavior regulation, aiming to provide a more comprehensive understanding of the underlying mechanisms governing cellular responses to biomaterials. By focusing on cell metabolic regulation, advanced materials have the potential to benefit a larger population in overcoming bone defects.

## 2 Mechanisms of bone regeneration

**FIGURE 1:**
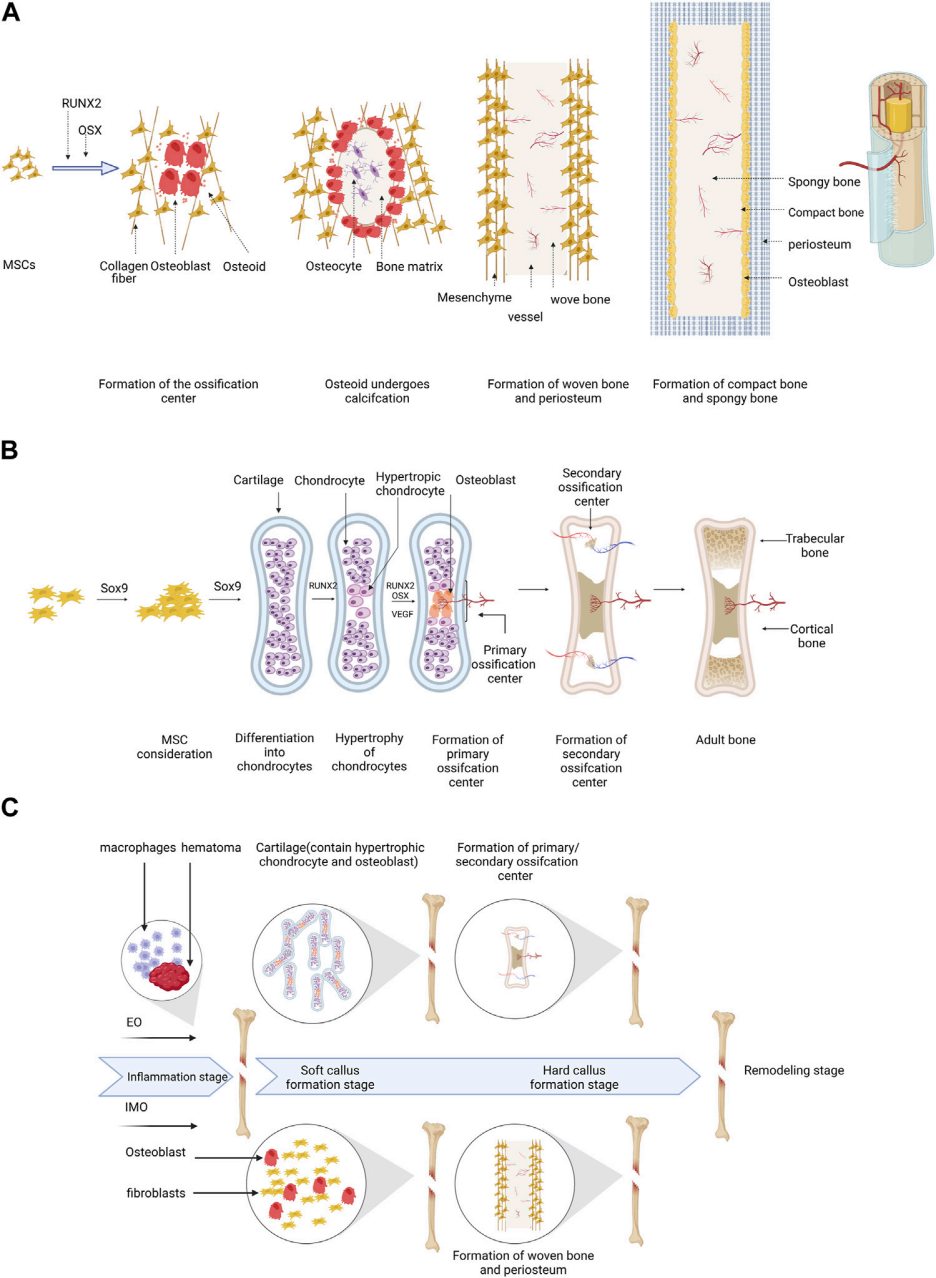
A schematic of the process of EO and IMO can be illustrated as follows: **(A)** IMO involves MSC differentiation into osteoblasts with Runx2 and Osterix assistance, ossification center formation, woven bone formation, and replacement of woven bone. **(B)** EO includes MSC transfer to chondrocytes with Sox9 involvement, hypertrophy, calcification, matrix degradation, primary and secondary ossification center formation, ossification center maturation, and adult bone formation. **(C)** The bone defect healing process generally consists of four stages, including inflammation, soft callus formation, hard callus formation, and remodeling. In the inflammation stage, hematoma and necrotic bone tissue are removed by macrophages, and the immune system is activated to fight infection. In the soft callus formation stage, fibroblasts and chondrocytes migrate to the site and form a soft callus, consisting of cartilage and collagen fibers. In the hard callus formation stage, osteoblasts produce new bone tissue, which mineralizes and hardens over time. In the remodeling stage, the bone tissue is further modified and reshaped to its original form by osteoclasts and osteoblasts. All the figures in this review created with BioRender.com.

Bone defects are characterized by shortages in the bone matrix, usually resulting from trauma or surgery, and frequently resulting in delayed union of bone, as well as dysfunction in the local area of the body (El-Rashidy et al., 2017). In the context of bone defect therapy, it is imperative to contemplate factors such as the anatomical position of the defect, the magnitude of adjacent tissue injury, and the overall health status of the body (Schemitsch, 2017). According to Haines et al. (2016), treatment efficacy is influenced by factors such as defect size and infection severity. Thus, a comprehensive consideration of multiple factors related to the defect is necessary for achieving better treatment in the clinical bone defect. Bone regeneration can occur via two pathways, intramembranous ossification (IMO) and endochondral ossification (EO) (Figure 1), both of which are vital for bone regeneration after injury. In brief, IMO increases osteoblast-related periosteum leading to periosteal thickening and calcification, which ultimately connect the fracture ends. EO initiates aseptic inflammatory response amidst the hematoma and bone marrow cavity, along with the surrounding milieu, creating fibrous, granulation, and transient cartilage tissues. This process then allows osteoblasts to invade and replace chondrocytes, leading to bone formation (Lopes et al., 2018). The process of bone repair subsequent to injury varies from physiological bone formation. (S. Wei et al., 2020) (Figure 1). Once the graft is fixed in place and fills the gap, IMO or EO are the primary modes of repair for bone defects. To repair bone defects, various materials have been designed based on different ossification strategies. Mineralized biomaterials (e.g., calcium phosphate-based ceramics) have been found to be effective activators of IMO pathways according to some studies (Gonzalez Diaz et al., 2018). In contrast to mineralized materials, naturally and synthetic polymers facilitate the process of EO pathway by enhancing cell attachment and subsequent differentiation. Despite being a well-documented observation, the precise mechanisms underlying the diverse osteogenic pathways induced by distinct biomaterials remain ambiguous (Bouler et al., 2017). Additionally, the mechanical support, cell adhesion, and nutrient exchange provided by scaffolds depend on their porosity and mechanical properties. These factors are critical for creating an ideal scaffold (Bian et al., 2013). The EO pathway is responsible for the majority of bone growth, as it triggers undifferentiated stem cells to differentiate into functional osteocytes through external factors like a mineralized platform, akin to the IMO pathway (Saul et al., 2023). Researchers are interested in stimulating EO for bone regeneration. Biomaterials stimulate bone regeneration by providing stimulatory via EO pathway, including progenitor cells and growth factors (Dang et al., 2017; Liu et al., 2018). Petersen demonstrated that a biomaterial-based solution, which mimics the natural extracellular matrix (ECM), can effectively induce EO repair of bone defects (Petersen et al., 2018). Furthermore, apart from biomaterials, Nilsson Hall and others (Hall et al., 2020) have discovered that multinodular structures formed by specific cells can repair bone defects via the EO pathway.

We have summarized recent advancements in the comprehension of metabolism in osteoblasts. Nevertheless, there is still much to learn about osteoblasts throughout differentiation, bone formation, and diseases. Osteoblast metabolism plays a crucial role in supplying energy and metabolites necessary for differentiation and activity. This knowledge advances our understanding of osteoblast biology and may guide innovative approaches for our patients with bone disorders.

## 3 Cell metabolic regulation in bone regeneration

### 3.1 The bone remodeling cycle

The skeleton is a versatile organ that provides support, protection, and levers for muscles. It also has metabolic, endocrine, and hematopoietic functions (DiGirolamo, Clemens, and Kousteni, 2012; Mera et al., 2017; Mosialou et al., 2017; Komori, 2020). The process of bone modeling and remodeling enables the skeleton, which is a dynamic and metabolic organ, to undergo constant “construction” and “reconstruction” (Mosialou et al., 2017). Bone resorption and formation are involved in bone modeling, they occur separately at different skeletal sites, causing significant changes in bone architecture. In contrast, the process of bone remodeling tightly links the resorption and formation of bone in space and time to maintain overall bone volume and structure.

The process of bone remodeling prevents brittle hyper-mineralized bone accumulation, releases stored calcium and phosphorus, and repairs skeletal damage (Manolagas, 2000; Resch et al., 2022). Osteoclasts are responsible for resorbing small regions of bone, with osteoblasts subsequently replacing them; this coordinated process of resorption and formation allows up to 10% of the skeleton to be renewed annually while preserving structural integrity. Various factors regulate such remodeling, with the pathways being uncovered through the investigation of rare bone diseases in families and animal models. In Part 3.3, we will provide a detailed introduction to the regulation of cell metabolism.

### 3.2 Types of cells involved bone remodeling cycle

Osteogenesis involves multiple cell types, such as osteoblasts, osteocytes, and chondrocytes (Ambrosi et al., 2019), which contribute to bone and cartilage formation and maintenance during homeostasis and injury. Osteoclasts, which are responsible for breaking down bone tissue, are derived from the hematopoietic lineage, whereas, on the other hand, other cell types involved in bone formation originate from different lineages (Salhotra et al., 2020).

Osteoblasts are the primary cells responsible for constructing bone tissue, which synthesize and release a variety of extracellular matrix proteins such as alkaline phosphatase. Multiple osteoblasts come together to form osteons, with calcium deposited as hydroxyapatite, along with type I collagen, to provide structural support for the skeleton (Long, 2011; Wagley et al., 2020). Osteoblasts specify the skeletal lineage through three stages of increasing differentiation: osteoprogenitors, preosteoblasts, and osteoblasts (Ambrosi et al., 2019). Commitment to osteoprogenitors is initially marked by the transcription factor SOX9, which also directs differentiation towards chondrocytes. RUNX2 expression in osteoprogenitors signals commitment to preosteoblasts, while WNT-β-catenin signaling trigger the osterix (OSX; also known as SP7) in preosteoblasts, determining their differentiation into osteoblasts. Finally, commitment to mature osteoblasts is signaled by both RUNX2 and OSX (Maeda et al., 2013). Preosteoblasts and osteoblasts, which are responsible for bone formation, exhibit significant differences in their energy metabolism. While preosteoblasts rely mostly on glycolysis for energy production, osteoblasts preferentially utilize oxidative phosphorylation (OXPHOS) in their mitochondria. This metabolic shift during the differentiation process enables the expression of genes and proteins specific to osteoblast function. In addition, osteoblasts possess a higher number of mitochondria than preosteoblasts to support their higher energy demands for bone formation (Karsenty and Wagner, 2002; Lee et al., 2017).

Due to limitations in the length of the article, this piece will solely concentrate on the metabolic control of osteoblasts, even though osteoclasts also have a substantial role in bone remodeling.

### 3.3 Metabolic regulation in osteoblast

#### 3.3.1 Regulation of catabolism and anabolism in osteoblasts

**FIGURE 2:**
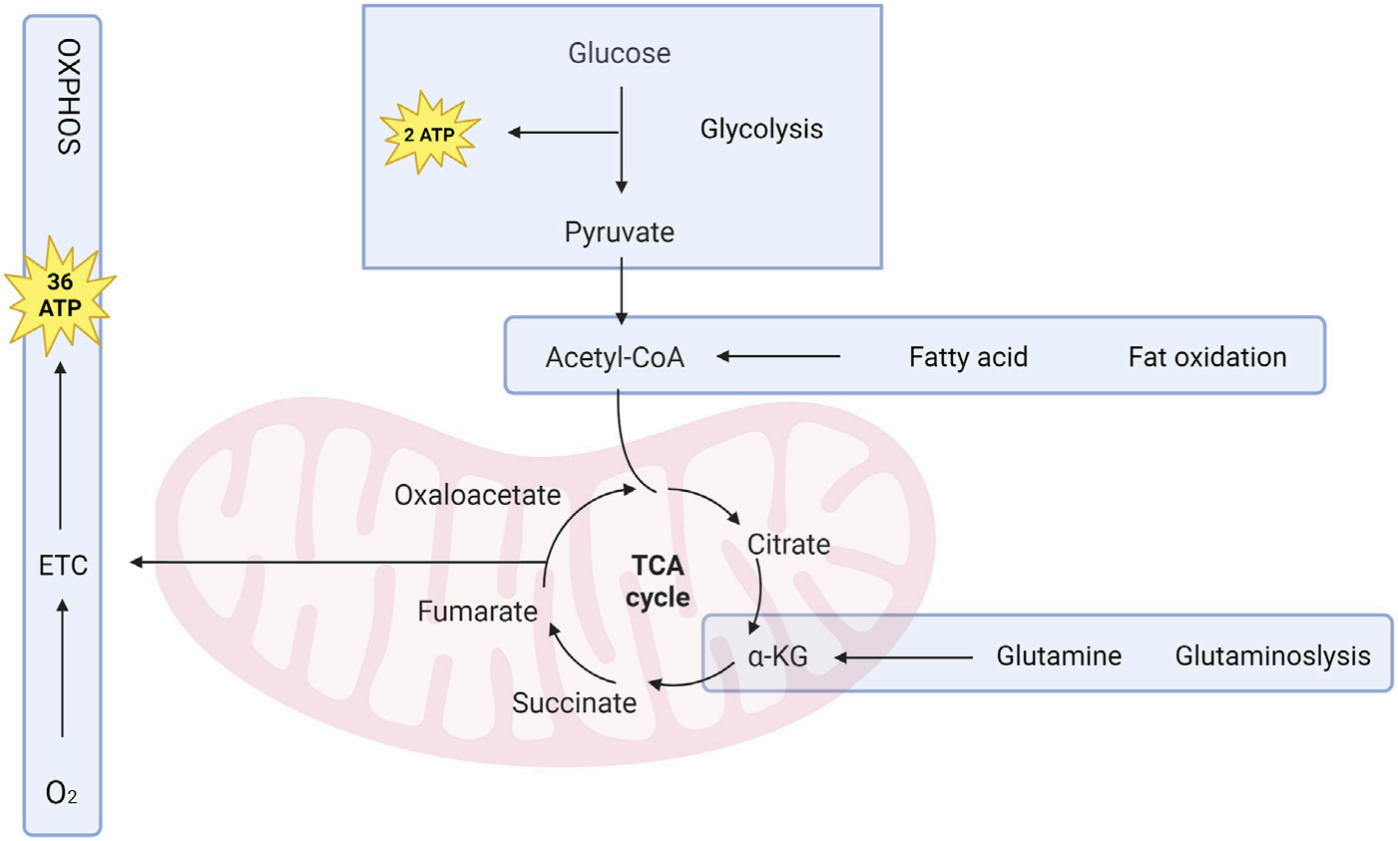
Osteoblasts rely on metabolic pathways to survive, proliferate, differentiate, and perform their functions. ATP, the primary energy source, is generated through glycolysis and cellular respiration. Glucose is converted to pyruvate through glycolysis, generating 2 ATP per glucose molecule. Pyruvate is then changed into acetyl-CoA, which enters TCA cycle to produce 36 ATP through OXPHOS. Fatty acids and glutamine can also be used as alternative fuel sources for the TCA cycle. AKG, derived from glutamine, is a primary energy source for Osteoblast.

The complex chemical reactions that take place within osteoblast, collectively known as cell metabolism, are characterized by a high degree of coordination between multiple enzyme systems. Osteoblasts use a complex metabolic process involving catabolism to break down nutrients and generate ATP for energy, and anabolism to create molecules needed for cellular activity. Glucose is a kind of important energy source, with glycolysis and OXPHOS being the main energy-producing pathways. The TCA cycle (Figure 2) generates electron carriers like NADH and FADH2, which donate electrons to the mitochondrial electron transport chain (mETC) for OXPHOS. Fatty acids and glutamine can also replenish the TCA cycle (C. Ma et al., 2019). To ensure metabolic stability, cells have formed highly adaptive mechanisms to control metabolic fluxes (Dempsey, 2018). Osteoblasts adjust their metabolic activity and pathways in response to hormones, which enable signals to be communicated between tissues. This adjustment is achieved by modulating gene expression, mRNA transcription, leading to changes in metabolic enzymes level. The metabolic adaptations that occur in response to cell signaling are tailored to support specific physiological functions. (Sivanand et al., 2018). Osteoblast progenitor cells can promote glucose uptake via glucose transporter 1 (Glut1) expression in response to osteogenic signals to fuel osteogenic differentiation. (Lee et al., 2018). Furthermore, there have been accounts of escalated glutamine intake and heightened expression of enzymes involved in glutamine breakdown. (Karner et al., 2015). Enzyme activity can be directly or indirectly affected by the presence of metabolic cofactors such as nonprotein chemical compounds (e.g., AKG) or metallic ions (e.g., Cu2+), which are necessary for enzyme function.

There is mounting evidence indicating that alterations in metabolic state have the potential to impact signaling pathways as well as gene expression. As metabolic flux can reciprocally influence the cellular state (Locasale and Cantley, 2011). In a previous publication, Van der Knaap conducted an extensive review on the potential impact of metabolic enzymes on cellular processes such as energy production, genetic expression, and chromatin modifications. This includes the use of metabolites as substrates for post-translational modifications (PTMs) of proteins, allowing cells to sense metabolite levels and to affect signal transduction pathways (van der Knaap and Verrijzer, 2016). For instance, N-linked glycosylation, folding of growth factor receptors require glycolytic flux, acetyl-CoA availability, offering a means to connect metabolite availability with growth factor-mediated signaling (Brunner and Finley, 2021). Furthermore, it is worth noting that metabolites have the potential to act as signaling molecules themselves, as demonstrated by the widely recognized ATP signaling. (Mikolajewicz et al., 2021). Other intermediate metabolites, such as alpha ketoglutarate (AKG). According to relevant experimental result, it can be inferred that, AKG can induce JNK phosphorylation in osteoblasts. It was observed that preconditioning of the cells with a JNK inhibitor resulted in the abolition of the increase in JNK phosphorylation, ALP activity induced by AKG, as well as the production of OPN and BSPII (Zurek et al., 2019).

While the core metabolic pathways remain unchanged, it is crucial to take into account the regulation of metabolism in the particular tissue-specific metabolic milieu. Variations in the microenvironment among different cells may include differences in the availability of nutrients such as amino acids and glucose, as well as fluctuations in the levels of oxygen, metabolites, and reactive oxygen species (ROS). (Litsios et al., 2018). The local cell environment can vary greatly in bone remodeling, where bone defect often disrupts the vascular network, leading to poor nutrient and the accumulation of ROS. Furthermore, cells possess the capability to modify their microenvironment by releasing intermediate metabolites, which can serve as signaling molecules or directly partake in physiological functions. (C. Ma et al., 2019). In the subsequent sections, the pathways and factors governing osteoblast metabolism will be presented.

#### 3.3.2 Key transcription factors of metabolic regulation in osteoblast

During osteoblast metabolic regulation, transcription factors are activated at specific moments to signal the necessary cues for specifying the osteoprogenitor’s functions as it changed into osteoblast. This section examines crucial transcription factors, including RUNX2, OSX, ATF4.

RUNX2 is a vital transcription factor involved in osteoblast development and bone formation, which acts by forming heterodimers with core binding factor subunits-β. Glucose is the primary nutrient for osteoblasts and is transported into these cells through Glut1. The expression of Glut1 precedes that of Runx2. The uptake of glucose promotes osteoblast differentiation by suppressing the AMPK-dependent proteasomal degradation of Runx2. It also supports bone formation by inhibiting another AMPK function. When glucose uptake is compromised, RUNX2 cannot induce osteoblast differentiation (Kong et al., 2021). However, raising blood glucose levels can restore collagen synthesis in Runx2-null osteoblasts and initiate bone formation in Runx2-deficient embryos. Additionally, RUNX2 favors the expression of Glut1. This feedforward regulation between RUNX2 and Glut1 determines the onset of osteoblast differentiation during development and the extent of bone formation throughout life (Kong et al., 2021). Runx2 is responsible for regulating the expression of several osteoblast genes, mainly including Spp1, and Bglap2, which encode osteopontin, and osteocalcin 2, respectively (Komori, 2019). As immature osteoblasts develop into mature osteoblasts in mice, the expression of Runx2 decreases. (Komori, 2010). When Runt-related transcription factor 2 is deleted, there is a total absence of osteoblasts and the expression of Spp1 and Bglap2 is lost. (Komori et al., 1997), highlighting the essential role of RUNX2 activity in early bone formation stage associated with osteoblast differentiation. Research utilizing a mature osteoblast promoter Col1a1 to conditionally knockout Runx2 has yielded inconsistent outcomes. Exon 4 Runt domain deletion did not cause phenotypic changes (Inada et al., 1999), while exon 8 deletion truncating Runx2 led to reduced bone formation (Takarada et al., 2013). Deletion of exon 9 in Runx2 has been linked with delayed maturation of osteoblasts. (Takarada et al., 2013). These findings align with the reduced expression of Runx2 in mature osteoblasts. (W. Zhang et al., 2022). To summarize the role of RUNX2 in metabolic regulation of osteoblast, RUNX2 initiates the differentiation of MSCs into preosteoblasts. In the perichondrium of endochondral bones, Ihh (Indian hedgehog) is required for the expression of Runx2. Preosteoblasts differentiate into immature osteoblasts when Runx2 induces the expression of Sp7 and canonical Wnt signaling. Furthermore, Runx2 and Sp7 also contribute to mature the osteoblasts. Proliferation of precursor cells that differentiate into osteoblasts is regulated by Runx2, which induces Fgfr2/3. The expression of Runx2, Sp7, hedgehog, Fgf, and Wnt signaling pathway genes is interrelated and mutually regulated (Komori, 2019; Hojo, 2023).

**OSX** (Osterix, also called Transcription factor Sp7), an essential osteoblast transcription factor, promotes the immature osteoblasts transfer into mature osteoblasts, and is critical for normal skeletal development (Liu et al., 2020). Its role in cell metabolism is mainly through regulating the expression of genes involved in energy metabolism, mitochondrial function, and glucose utilization. Several studies have shown that Osterix can promote mitochondrial biogenesis and function in osteoblasts (Shahi et al., 2017). It does so by activating the expression of genes involved in oxidative phosphorylation and mitochondrial respiration, which results in increased ATP production and enhanced cellular metabolism (Pierce et al., 2022). Osterix also regulates the expression of genes involved in glucose metabolism, including GLUT1, a glucose transporter that facilitates glucose uptake and utilization in osteoblasts (Kong et al., 2021). In mice, higher OSX expression during osteoblast maturation is inversely correlated with Runx2 expression. When OSX is absent, the formation of bone is impaired, and there is a loss of osteoblast gene expression, such as Sparc and Spp1 (Nakashima et al., 2002), resulting in a failure of bone formation. Inactivation of OSX postnatally caused disturbed bone formation, porous cortical bone, osteoblast dysfunction, and reduced expression of osteoblast genes, Sost and Dkk1 (X. Zhou et al., 2010). OSX plays a crucial role in the maturation of osteoblasts, and its expression is downstream of RUNX2. Like RUNX2, it is involved in the differentiation of preosteoblasts into immature osteoblasts and immature osteoblast to mature cell, which eventually contribute to the formation of mature osteoblasts (Liu et al., 2020; Wang et al., 2023).

ATF4 (Activating transcription factor 4) is a transcription factor with a leucine zipper and a member of the CREB family that initially discovered as a nuclear binding protein that is highly expressed in osteoblasts. ATF4 plays a key role in regulating cellular metabolism, particularly in response to stress conditions. It regulates several metabolic pathways including amino acid metabolism, lipid metabolism, and glucose metabolism. ATF4 regulates the expression of genes involved in the pentose phosphate pathway, which generates NADPH, an important cofactor for the biosynthesis of fatty acids and nucleotides. ATF4 also regulates the expression of genes involved in the regulation of glucose homeostasis, such as Glut1 and HK2, which is responsible for glucose uptake in cells (Mahor et al., 2022). Moreover, ATF4 is a crucial regulator of mitochondrial function and oxidative metabolism. It promotes mitochondrial biogenesis, regulates the expression of genes involved in mitochondrial respiration, and enhances the activity of the electron transport chain, thereby increasing ATP production (Quirós et al., 2017). ATF4 also regulates osteoblast differentiation by promoting the synthesis of type I collagen through post-translational transcription modification. Additionally, it enhances osteogenesis based on producting complex with SATB2, which cooperatively interacts with RUNX2 and promotes its activity. Furthermore, Atf4-knockout mice have shown delayed bone trabeculae formation, resulting in lower bone mass, strength. (Jing et al., 2015; Komori, 2020). A Both ATF4 and FIAT, which mainly appeared in osteoblasts, and ATF4 activity is regulated by FIAT, which suppresses its effects (Ebert et al., 2022), Moreover, ATF4 plays a crucial role in regulating glucose metabolism in osteoblast. The study showed that ATF4 regulates the expression of several key genes involved in glucose metabolism, including Glut1 and HK2, which are responsible for glucose uptake and phosphorylation (X. Yang et al., 2004). In summary, ATF4 is a key regulator of bone metabolism. It promotes osteoblast differentiation by increasing the synthesis of type I collagen, and cooperates with SATB2 to enhance RUNX2 activity, thereby promoting osteogenesis. Moreover, ATF4 plays a vital role in regulating glucose metabolism in osteoblasts by controlling the expression of genes such as Glut1 and HK2. Additionally, it regulates genes involved in mitochondrial respiration and boosts the activity of the electron transport chain, leading to an increase in ATP production.

Other transcription factors, such as GLI1, GLI2, AP-1, TAZ, and HOX11, play crucial roles in osteoblast differentiation stimulation. In contrast, TWIST, HOXA2, HAND2, and GLI3 suppress osteoblast differentiation. Additionally, FIAT and FOXO can regulate ATF4 function, which in turn affects the number of osteoblasts (Salhotra et al., 2020).

Paracrine, autocrine, and endocrine factors such as bone morphogenetic proteins (BMPs), growth factors, and hormones can modulate osteoblast differentiation and maturation, with PTH and BMPs having effects closely linked to Wnt signaling activation. (Ramesh et al., 2021). Fully differentiated osteoblasts co-express with type I collagen, essential for bone matrix synthesis and mineralization. They can release regulators of matrix mineralization, including osteonectin, as well as RANKL and the PTH receptor. At lifespan end, osteoblasts differentiate into osteocytes embedded in lining cells which can protect bone. Specific molecules, such as sclerostin, regulate bone formation and phosphate metabolism (S. Vimalraj, 2020; Uda et al., 2017).

#### 3.3.3 Key signaling pathways of metabolic regulation in osteoblast

Understanding the cellular and molecular mechanisms underlying bone regeneration is crucial, despite significant advancements made in this field. Several studies have established the participation of important signaling pathways, including Wnt, Notch, BMP/TGF-β, PDGF, IGF, FGF, Ca2+, JNK, MARK and mTOR/S6K1/S6 pathway (Majidinia et al., 2018).

The Wnt signaling pathway regulates cell fate determination, proliferation, and differentiation. In the canonical pathway, After binding to LRP5 complex, extracellular Wnt ligands activate intracellular disheveled (DSH), which prevents the degradation of 𝛽-catenin by the axin-GSK3-APC(axin, glycogen synthase kinase 3 (GSK3).H ence it can be infered that, 𝛽-catenin translocates to the nucleus, in which it heterodimerizes with lymphoid enhancer-binding factor/T factor to regulate gene transcription, including that of RUNX2, crucial in osteoblast differentiation. (James, 2013). In the non-canonical Wnt signaling pathway, The Wnt3a signaling pathway activates mTORC2 and AKT via LRP5 and RAC1, promoting hexokinase 2 (HK2) and phosphofructokinase 1 (PFK1). This enhances aerobic glycolysis, also known as the Warburg effect, and encourages osteogenic differentiation (Esen et al., 2013). Moreover, the Wnt/mTORC1 signaling pathway increases glutamine consumption through the tricarboxylic acid cycle, providing energy and promoting the integrated stress response (ISR) via general regulatory repressor protein kinase 2 (GCN2), which boosts amino acid supply, tRNA aminoacylation, and gene expression related to protein folding( Karner et al., 2015).

Researchers suggest that Wnt signaling pathway functions as an upstream regulator of mTOR to facilitate osteogenic differentiation (Maeda et al., 2019). This part of the review will only focus on the role of mTOR/S6K1/S6, MARK and JNK signaling pathways in bone regeneration.

### 3.4 mTOR/S6K1/S6 pathway

Mammalian target of rapamycin (mTOR) exert a critical function in numerous cellular behavior such as cell growth, survival, proliferation, and motility, making it a vital mitogenic signaling pathway. (Majidinia et al., 2018). The mTOR kinase is responsible for two distinct complexes, namely ,mTORC1 and mTORC2. mTORC1 is responsible for regulating protein, lipid, and nucleotide synthesis as well as inhibiting autophagy to promote cell growth and regulating metabolism. In contrast, mTORC2 controls cell survival according to the study (Saxton and Sabatini, 2017). The regulation of its differentiation has been linked to both mTORC1 and mTORC2. (Chen and Long, 2015). Stimulation of osteoblast differentiation occurs when mTORC1 is activated by various bone anabolic signals (e.g., IGF-1, Wnt3a, (Zurek et al., 2019). The two primary substrates of mTORC1 are S6 kinase 1(S6K1) and 4E-BP1 (Saxton and Sabatini, 2017). Activation of S6K1 by mTORC1 results in the phosphorylation of the S6 in the 40S ribosomal subunit. This phosphorylation facilitates important biosynthetic pathways associated with cell growth, and it also disrupts the inhibitory association between eIF4F and 4E-BP1. This enables effective 5′cap-dependent translation of mRNAs associated with cell cycle. While 4E-BP1 primarily regulates proliferation in various mammalian cells, often in an independent manner (Dowling et al., 2010). This leads to an increase in the production of proteins, which are essential for cellular functions such as metabolism, growth, and proliferation. mTOR also regulates cell metabolism by modulating the activity of various metabolic enzymes and pathways. For example, mTOR stimulates glycolysis (the breakdown of glucose) by promoting the expression of glucose transporter proteins and the activity of key glycolytic enzymes (Cheng et al., 2014).

AKG has also been shown to promote protein synthesis via mTORC1 in recent studies (Cai et al., 2016). Recent research indicated that AKG promotes the growth of osteoblasts by inducing the phosphorylation of mTOR, S6K1 without affecting 4E-BP1 phosphorylation. AKG accomplishes this through the mTORC1 related control of S6K1/S6 axis (Zurek et al., 2019). Moreover, recent research suggests that the transition from immature to mature osteoblasts requires mTORC1. (Ahmadi et al., 2022).A ctivating Akt, a crucial mediator of insulin/PI3K signaling, is the primary function of mTORC2. Upon activation, Phosphorylation and inhibition of key targets like FoxO1/3a, GSK3b, and mTORC1 inhibitor TSC2 by Akt promote cell growth, proliferation, and survival. Another AGC-kinase called SGK1, which regulates ion transport and cell survival through phosphorylation, is also activated by mTORC2. (Saxton and Sabatini, 2017; Rhoads and Anderson, 2020).

### 3.5 MAPK pathway

The MAPK is a significant factor in connecting the cell surface to nucleus, thereby regulating various cellular processes such as proliferation, differentiation, migration, and cell death (Daigang et al., 2016). The MAPK is vital in bone formation as it has the ability to communicate with various molecular. (Daigang et al., 2016). Researchers examined the involvement of MAPK signaling, particularly the ERK1/2, which can be regrade as a crucial regulator of the transcriptional behavior that facilitate this process. (Zurek et al., 2019). Studies have shown that ERK1/2 phosphorylates RUNX2 (Ge et al., 2009), which enhances its transcriptional activation. The function of ERK signaling in osteoblast differentiation differs among cell types. According to Matsuguchi’s study (Matsuguchi et al., 2009) the inhibition of MEK1/2, which can either enhance or impede matrix mineralization in different cell lines. Moreover, The MAPK pathway is vital for bone repair after injury because it transduces signals from growth factors and adhesion molecules such as EGF, IGF, and α2/5 integrins. A study on rats found that dual leucine zipper kinase (DLK), a component of this pathway, was temporarily upregulated during the post-fracture period. (Majidinia et al., 2018).t he c-Jun NH2-terminal kinases (JNK) pathway, which is an evolutionarily conserved subgroup of MAPK. Several studies have indicated that the JNK pathway participates in osteoblast differentiation. For instance, Matsuguchi et al. (2009) found that JNK activity is critical for ATF4 expression, which is necessary for late-stage osteoblast differentiation. ATF4 is crucial for bone regeneration. Inactivation of Smurf1, led to the accumulation of MEKK2 and subsequent JNK activation, resulting in increased bone mass. Moreover, AP-1 transcription factor was found to be activated by JNK. It is worth noting that several genes involved in osteoblast differentiation, including RUNX2, SPP1, BGLAP and COL1A1, contain AP-1 binding sites in corresponding promoter region (Lee et al., 2018). In Zurek A’s study (Zurek et al., 2019), JNK phosphorylation in osteoblasts by AKG was shown for the first time in a study. The study also found that pretreatment with a JNK inhibitor eliminated the AKG-induced higher ALP activity, production of BSPII and OPN. In summary, these findings indicate that the MAPK pathway and JNK pathway (subgroup of MAPK) is crucial in metabolic regulation of osteoblast. They regulate metabolic processes in osteoblasts through its control of Runx2 differentiation and bone formation. MAPK signaling has been shown to promote the expression and activity of Runx2, which in turn promotes the expression of genes involved in osteoblast differentiation. In addition to its effects on Runx2, the MAPK pathway also regulates metabolic processes in osteoblasts through its control of AMP-activated protein kinase (AMPK), a key regulator of cellular energy homeostasis. Which in turn leads to an increase in cellular ATP levels and a decrease in AMPK-dependent metabolic processes, such as glucose uptake.

### 3.6 AMPK pathway

AMP-activated protein kinase (AMPK) is a critical regulator of energy metabolism and has been shown to play a role in osteoblast function and bone formation (Steinberg and Kemp, 2009). Activation of AMPK in osteoblasts has been shown to promote osteoblast differentiation and mineralization, while inhibiting osteoclast differentiation and bone resorption. One study showed that activation of AMPK in osteoblasts by metformin increased bone mass and strength in mice by promoting osteoblast differentiation and reducing osteoclast activity (Molinuevo et al., 2010). In addition, AMPK activation in osteoblasts increased the expression of bone matrix proteins, such as collagen type I and osteocalcin, which are essential for bone formation and mineralization (Shah et al., 2010). Moreover, recent studies suggest that AMPK signaling may also be involved in the regulation of glucose metabolism in osteoblasts. AMPK activation in osteoblasts has been shown to increase glucose uptake and glycolysis, while inhibiting gluconeogenesis and has also been shown to regulate the expression of key genes involved in glucose metabolism, such as Glut1 and HK2 (Vogel et al., 2021; J; Wei et al., 2015).Together, these studies suggest that AMPK signaling plays a crucial role in osteoblast function and bone formation, and that activation of AMPK in osteoblasts is important for bone nodule formation *in vitro* and the maintenance of bone mass *in vivo* further supporting a role for AMPK signaling in skeletal physiology.

We have reviewed the natural fracture repair and regeneration process and discussed the involvement of key molecular signaling pathways. Understanding these pathways can lead to targeted interventions to enhance bone regeneration. As mentioned previously, the Wnt signaling pathway regulates mTOR upstream. AKG has been shown to promote protein synthesis through the mTORC1-dependent pathway, which regulates protein, lipid synthesis while inhibiting autophagy and promoting cell growth through metabolic regulation. Additionally, mTORC2 controls cell proliferation and survival, ultimately facilitating osteogenic differentiation. By comprehending the pathways and essential factors for osteoblast differentiation, bioactive polymers based on AKG can be designed to enable clinicians to promote bone regeneration by utilizing endogenous MSCs to drive osteoblast differentiation. It is valuable to research the osteoblast lineage, and an increased understanding of it through new technologies can provide support for research and clinical treatment.

## 4 Biomaterials involved in the metabolic regulation for bone regeneration

### 4.1 Materials with advanced physicochemical characteristics

Currently, clinically widely used bone regeneration materials include metals (such as magnesium, titanium), ceramics (such as hydroxyapatite), and polymers (such as polyether ether ketone, collagen). However, challenges remain regarding the degradability of metals and synthetic polymers, the compatibility of bone regeneration processes (Yang L. et al., 2020; Zhao et al., 2021), and insufficient mechanical properties of bioceramics and natural polymers (Dejob et al., 2021; Pan et al., 2021). Therefore, a preferred strategy in the field of bone tissue engineering is combining multiple materials and their advantages to prepare scaffolds with bioactivity, suitable porosity, and excellent mechanical properties.

**FIGURE 3:**
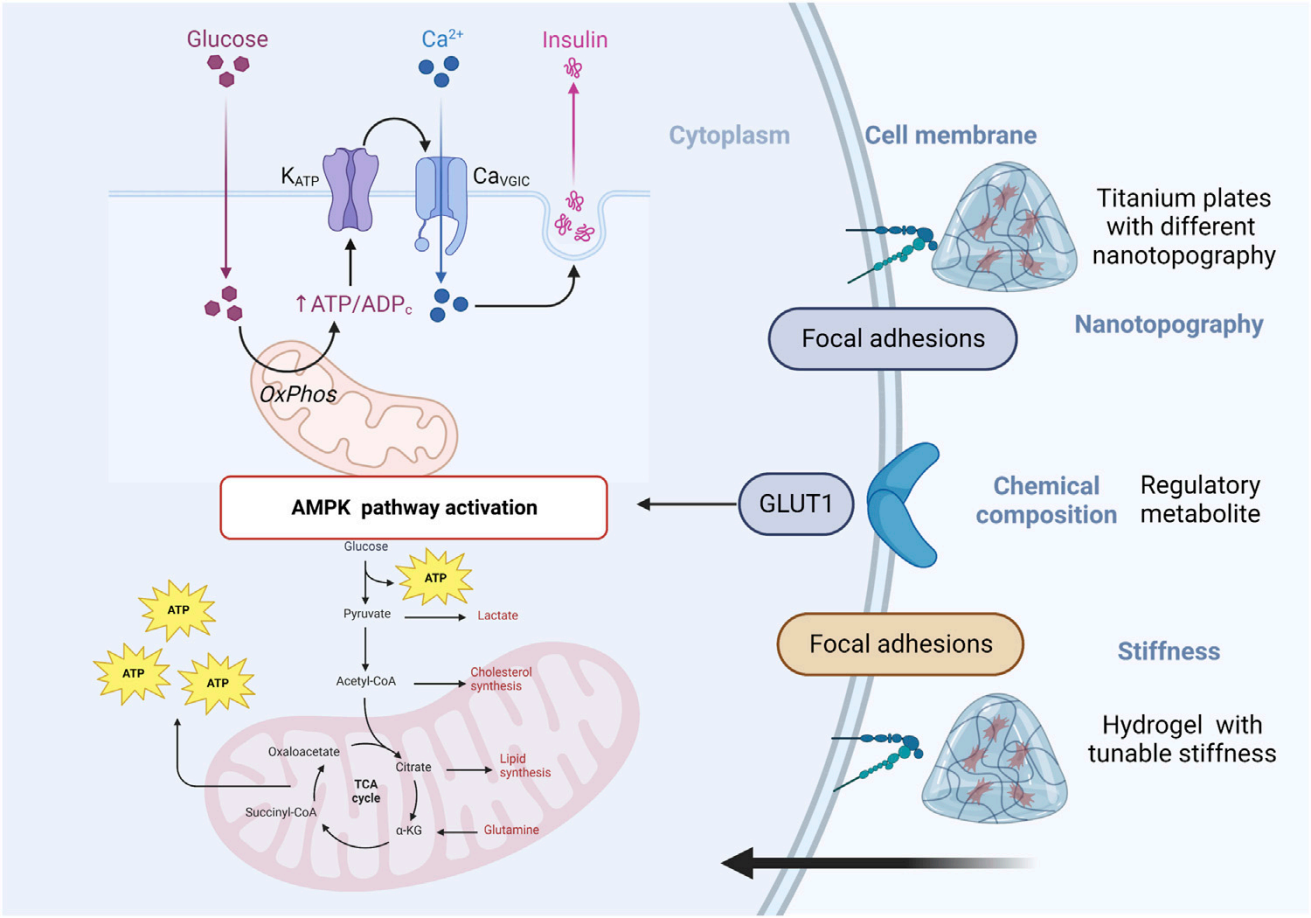
Schematic of materials with advanced physicochemical characteristics can influence cell metabolism.

Materials with advanced physicochemical characteristics can have a significant impact on cell metabolism, both in terms of cellular behavior and overall cellular health. Some of the ways in which materials can influence cell metabolism. They have been designed to mimic the extracellular matrix of bone tissue and can influence osteoblast metabolism by altering cell adhesion and proliferation, protein adsorption and signaling, oxygen and nutrient diffusion, for instance, Materials with specific surface properties, such as nanotopography, stiffness and chemical composition, can influence the adhesion and proliferation of cells (Figure 3). These factors can alter the activation of signaling pathways that regulate cell metabolism, leading to changes in cell growth, differentiation, and survival (Zuo et al., 2013). The properties of a material can affect the adsorption of proteins from the surrounding environment, which in turn can influence cell signaling and metabolism. For example, Materials with advanced physicochemical characteristics can promote osteoblast differentiation and mineralization, This process is regulated by a number of metabolic pathways, including the TCA cycle, glycolysis, and oxidative phosphorylation (C. Zhou et al., 2023). Furthermore, the metabolic activity of osteoblasts can affect the expression of key transcription factors and signaling pathways involved in osteoblast differentiation and bone formation such as BMP-2 which can promote osteoblast differentiation and mineralization via MAPK pathway. In summary, materials with advanced physicochemical characteristics can affect osteoblast metabolism and regulate key pathways involved in osteoblast differentiation and bone formation.

### 4.2 Materials binding exogenous stimuli

Materials that bind exogenous stimuli can have a significant impact on cell metabolism by responding to external cues and modulating cellular behavior. Some examples of materials that bind exogenous stimuli include hydrogels that respond to changes in temperature or pH, nanoparticles that respond to magnetic or light fields, and surfaces that respond to electric fields (Yang et al., 2020). Promoting bone regeneration through physical interventions and exogenous stimuli (e.g., light, heat, electricity, and magnetic fields), has garnered significant attention as an area of interest. Clinical therapies involving exogenous stimulation have been shown to stimulate bone tissue growth, accelerate bone regeneration, and alleviate patient pain (Thompson et al., 2016). Integrating advanced material science and exogenous stimulus intervention to regulate and control the different stages of bone regeneration holds great promise for enhancing the efficiency of bone defect repair.

#### 4.2.1 Photothermal stimulation

Photothermal therapy (PTT) has recently emerged as a non-invasive, controllable approach for tissue regeneration and tumor elimination due to its high penetration ability. Thermosensitive polymers usually experience a so-gel transition when the temperature changes, which leads to modifications in their configuration. During critically low solution temperatures, water molecules form hydrogen bonds with polar groups, causing the hydrogel to become soluble. When the temperature rises above the lower critical solution temperature, polymer chains shrink and become hydrophobic and insoluble. (Onaca et al., 2009). Yang et al. developed microcarriers made of poly( N-isopropylacrylamide) which can swell when the temperature increases. These microcarriers can release encapsulated drugs when the temperature in the joint cavity rises due to exercise or osteoarthritis. (Yang et al., 2020).Other studies have found that mild heat (40–45°C) induced through PTT can promote bone tissue regeneration. Zhang et al. (2019) utilized porous alloy of gold-palladium (AuPd) nanoparticles to induce mild localized heating via near-infrared laser irradiation, which effectively stimulated cell proliferation and bone regeneration. Transplantation of rat bilateral skull full-thickness defects, which had an 8 m diameter, resulted in new bone formation covering 97% of the defect area after 6 weeks. Exogenous stimuli can regulate drugs or growth factors, as demonstrated by studies that the introduction of heat-activated, dimer-dependent transgene expression systems into MSCs, and the use of light-induced mild high temperature to induce the release of biologically active BMP-2 which can promote osteoblast differentiation and mineralization via MAPK pathway from the dimer (Sanchez-Casanova et al., 2020). The release and expression of BMP-2 is mainly dependent on the action of light and heat. Therefore, manipulating exogenous stimuli provides a potential solution for the controlled release of bioactive substances.

#### 4.2.2 Electrical stimulation

The application of exogenous electrical stimulation has been shown to promote the proliferation and differentiation of bone cells (Maharjan et al., 2020). The appropriate introduction of electrical stimulation also holds great promise for metabolism regulation in osteoblast. Electrical stimulation can increase the activity of osteoblasts by altering their cellular metabolism. When a bone is subjected to an electric field, the movement of charged particles (ions) across the cell membrane creates an electric current within the cell. This current can stimulate some metabolic pathways within the cell, leading to an increase in cellular activity and the production of proteins and other molecules necessary for bone growth (Huang et al., 2019). Electrical stimulation can stimulate the production of BMP, IGF-1 and TGF-β, which are critical for bone growth and remodeling (Cui et al., 2020). Electrical stimulation can also increase the expression of genes involved in bone formation, such as collagen type I and osteocalcin (Huang et al., 2019). These genes encode proteins that are essential for the formation and mineralization of bone tissue.

#### 4.2.3 Magnetic stimulation

By activating cell surface receptors and their associated signaling pathways, exogenous magnetic stimulation can increase cell activity and facilitate integration of the scaffold. Go et al. conducted a study on knee cartilage regeneration using a magnetic adipose-derived microcarrier made of PLGA. Additionally, they designed a microrobot system that includes an electromagnetic actuation system and a magnet for fixation (Go et al., 2020). M oreover, a magnetic implant system was developed for stem cell-based knee cartilage repair, which includes magnetic microcarriers, a portable magnet array device, and a paramagnetic implant. This system was fabricated more recently (Go et al., 2021). Magnetic stimulation also can lead to an increase in calcium content, new bone density, and acceleration of bone healing. The magnetic scaffolds substantially activated the BMP-2 gene expression of cells, This, in turn, resulted in a significant elevation of Smad1/5/7 phosphorylation in response to the magnetic cues. Additionally, the scaffolds synergistically activated the p38, ERK1/2, and JNK pathways (Yun et al., 2016). The utilization of a magnetic scaffold in conjunction with an external static magnetic field shows promise in the field of bone regeneration engineering. The static magnetic field and magnetic nanoparticle composite scaffold synergistically promoted osteoblasts by activating the integrin signaling pathway and promoting angiogenic responses (Yun et al., 2016).

### 4.3 Materials with metabolic regulators

The influence of osteoblast metabolism on signaling and gene expression can be applied to the field of bone regeneration. This is evidenced by the significant impact that material cues have on cell behavior in biomaterials designed to replicate the tissue environment. (Crowder et al., 2016). Therefore, deliberate material design may enable the modulation of cellular metabolic status and the controlled release of endogenous metabolic modulators from biomaterials.

#### 4.3.1 Metal ions

Metal ions can have a significant impact on osteoblast metabolism, which is the metabolic activity of the cells responsible for bone formation. Metal ions play a crucial role as cofactors in metabolic enzyme activity modulation. Therefore, biomaterials doped with ions, such as Ca^2+^, Mg^2+^, Zn^2+^, Co^2+^, and Cu^2+^, which act as enzyme cofactors or indirectly impact enzyme activity by substituting key ion cofactors, have immense potential to regulate metabolism and facilitate controlled cell function in the context of bone regeneration. Zinc ions have been shown to upregulate the expression of genes involved in bone formation, such as Runx2, collagen I and osteocalcin (Vimalraj et al., 2019). Scientists also found that Zinc scaffolds have precise control over their pore structure. When the size of the pores increased, the scaffold became weaker and corroded faster. However, cells were able to stick and grow better on the scaffold after an *ex vivo*treatment, and it was shown to be biocompatible. Additionally, as the scaffold became more porous, it exhibited potent antibacterial properties. Therefore, Zinc scaffolds show great potential for orthopedic applications. (Cockerill et al., 2020). The most prominent instance involves metal ions, such as Co^2+^, Cu^2+^, and Mn^2+^, that competitive substitution of iron at the active site of enzymes containing iron, such as prolyl hydroxylase domains (PHDs), can lead to the stabilization and activation of HIF-1α. Cadmium ions can induce oxidative stress and reduce the activity of antioxidant enzymes in osteoblasts, leading to cellular damage and impaired bone formation. A bioactive glass doped with Co^2+^ was developed to release Co2+ in a controlled manner. This led to a concentration-dependent increase in HIF-1α activity, as expected, resulting in improved survival of hMSCs and increased expression of VEGF in these cells (Azevedo et al., 2015). The inclusion of Co2+-doped bioactive glass particles considerably improves the generation of alkaline phosphatase and calcium deposition, indicating significant progress in bone formation and cell growth by secretion of BMP-2. (Quinlan et al., 2015). Similarly, the incorporation of Cu2+ into biomaterials such as bioactive glass and graphene-based composites has been shown to promote angiogenesis and osteogenesis by increasing the secretion of VEGF and BMP-2, leading to the activation of HIF-1α and promote osteoblast differentiation and mineralization via MAPK pathway. (Zhang et al., 2019; Liang et al., 2021). Scientists discovered that rapid metabolism triggered by BMP-2 is crucial for osteogenesis. Inadequate stimulation causes low-dose BMP-2 to be ineffective. They found that Mg^2+^ acts as an “energy propellant” that boosts bioenergetic levels to support osteogenesis, leading to enhanced osteoinductivity of BMP-2. Based on this discovery, they developed microgel composite hydrogels as a low-dose BMP-2/Mg^2+^ delivery system (Lin et al., 2022).

#### 4.3.2 Bioactive molecules

In recent years, bone tissue engineering scaffolds combined with microcarriers. Various microcarriers have been developed for controlled cell metabolism and bone regeneration including growth factors, drugs, bioactive peptides, genes, and cells (Ding et al., 2022).

**Growth factors** play a crucial regulatory role in bone formation, remodeling, and regeneration, with a primary focus on enhancing the biological functions of bone grafts, including the recruitment of endogenous stem cells, migration of endothelial cells, and promotion of osteogenic differentiation (Martino et al., 2015). Loading different types of growth factors during distinct material action cycles can accurately replicate the bone regeneration process and lead to better functional performance *in vivo* as the required growth factors vary at different stages of bone regeneration. For instance, fibroblast growth FGF-2 is prominently expressed in the initial stages of bone healing, while BMP-2 is widely expressed throughout the process of osteogenic differentiation. Yin et al. (2018) incorporated BMP-2 into polylactic acid-polyethylene glycol-polylactic acid microcapsules and immobilized FGF-2 on their surface, and quantified bone volume and density in the defect area by micro-CT after 12 weeks of calvarial defect transplantation in rats. The research conducted by them demonstrated that the release of FGF-2 followed by BMP-2 was more beneficial for bone regeneration and new bone formation than the reverse order of release. Both FGF-2 and BMP-2 are essential growth factors that play crucial roles in promoting bone formation, remodeling, and regeneration. These growth factors achieve their functions through the activation of various metabolic regulation pathways, including MAPK the canonical Wnt pathway. The MAPK and Wnt pathway are known to stimulate the differentiation of osteoblasts, leading to the formation of new bone tissue resulting in increased bone density and strength. By regulating these metabolic pathways, FGF-2 and BMP-2 have been shown to contribute significantly to bone healing and regeneration processes.

**Drugs and bioactive peptides** are significant in the repair of bone defects by facilitating controllable and sustainable delivery through rational material structure design promoting cell metabolism when combined with microcarriers. Drug delivery microcarriers are typically made using natural or synthetic polymers. These microcarriers encapsulate the drugs or bioactive factors to increase bioavailability and provide a sustained release with a constant drug plasma concentration (Ding et al., 2022). Additionally, controlling the porosity and pore structure of the microcarriers are important properties. In a recent study, Yang et al. developed intelligent microcarriers that can shrink or swell in response to pathological triggers, providing drug release switches for treating osteoarthritis (Yang et al., 2020). A case in point is the encapsulation of BMP-2 into bovine serum albumin nanoparticles to preserve its biological activity (Li et al., 2015).B ovine serum albumin nanoparticles, polycaprolactone-polyethylene glycol copolymer, and dexamethasone (DEX) were electro spun to create dual drug-loaded nanofiber scaffolds. *In vitro*studies have shown that the scaffold effectively sustained the biological activity of both DEX and BMP-2, with DEX primarily released during the initial 8 days and BMP-2 gradually released over a period of 35 days. The loading and controlled release of DEX exhibited a stronger effect on bone regeneration. Additionally, alendronate (ALN) was incorporated into the scaffold with BMP-2, as it prevents the recruitment and differentiation of osteoclasts and binds to growth factors, releasing them sequentially. The ALN was encapsulated in biodegradable microspheres, and it was observed that BMP-2 was released in the early stage, while ALN was continuously released after 2 weeks, resulting in significantly improved bone regeneration 8 weeks after defect implantation (D. Lee et al., 2021). In summary, it has been demonstrated that materials that incorporate drugs such as alendronate and bioactive peptides like BMP-2 can exert significant effects on metabolism and regulate key pathways involved in osteoblast differentiation and bone formation. Specifically, these materials have been shown to activate pathways such as the MAPK and JNK pathways, which play critical roles in the regulation of osteoblast function and bone remodeling.

**Gene-based therapy** provides an alternative approach to bone regeneration, and various methods have been explored to prepare gene-/interfering RNA-activated scaffolds for bone tissue engineering, such as plasmid DNA, viruses, RNA transcription, or interfering RNA-activated scaffolds (Laird et al., 2021). For instance, polyethyleneimine (PEI) complexes were employed to prepare two types of plasmid DNA that encode for BMP-2and FGF-2. These plasmid DNA constructs were then loaded onto PEI collagen scaffolds for use in bone regeneration studies. Upon analysis, it was found that the PEI collagen scaffolds co-implanted with BMP-2 and FGF-2 plasmid DNA demonstrated significantly enhanced bone regeneration compared to scaffolds loaded with plasmid DNA alone. Specifically, the incorporation of both BMP-2 and FGF-2 plasmid DNA in the PEI collagen scaffolds resulted in a synergistic effect that promoted osteoblast differentiation and proliferation, ultimately leading to the formation of new bone tissue. These findings highlight the potential utility of using PEI complexes to deliver plasmid DNA encoding growth factors such as BMP-2 and FGF-2 for improved bone regeneration in tissue engineering applications. (Khorsand et al., 2017). Integrating biological strategies and stem cell technology into advanced material design has garnered significant clinical attention for developing potential bone graft scaffold materials. In cases of large bone defects, exogenous stem cell-based bone grafting can supplement the inadequate number of endogenous stem cells required for bone regeneration. Scaffold-mediated stem cell cultivation shows stronger application potential compared to simple stem cell injection or direct implantation. (Du et al. (2015) used methacrylamide gelatin as a scaffold for bone marrow MSCs and observed higher cell survival rates and more efficient induction of osteoblast differentiation within 14 days compared to osteogenic medium. Additionally, continuous delivery of BMP-2 and VEGF has great potential in increasing the number of MSCs required for bone defect treatment (Dashtimoghadam et al., 2020).

In summary, it has been demonstrated that a synergistic approach combining the addition of endogenous or exogenous cells and the regulation of scaffold structure can effectively promote bone regeneration through the activation of various metabolic regulation pathways. By incorporating cells into the scaffold structure, such as osteoblasts or MSCs, the scaffold can provide a suitable environment for cell growth and differentiation, which is critical for bone regeneration. Additionally, the regulation of scaffold structure can impact the activity of metabolic pathways, such as the MAPK and canonical Wnt pathways, which play critical roles in osteoblast differentiation and bone formation.

#### 4.3.3 Regulatory metabolite

**FIGURE 4:**
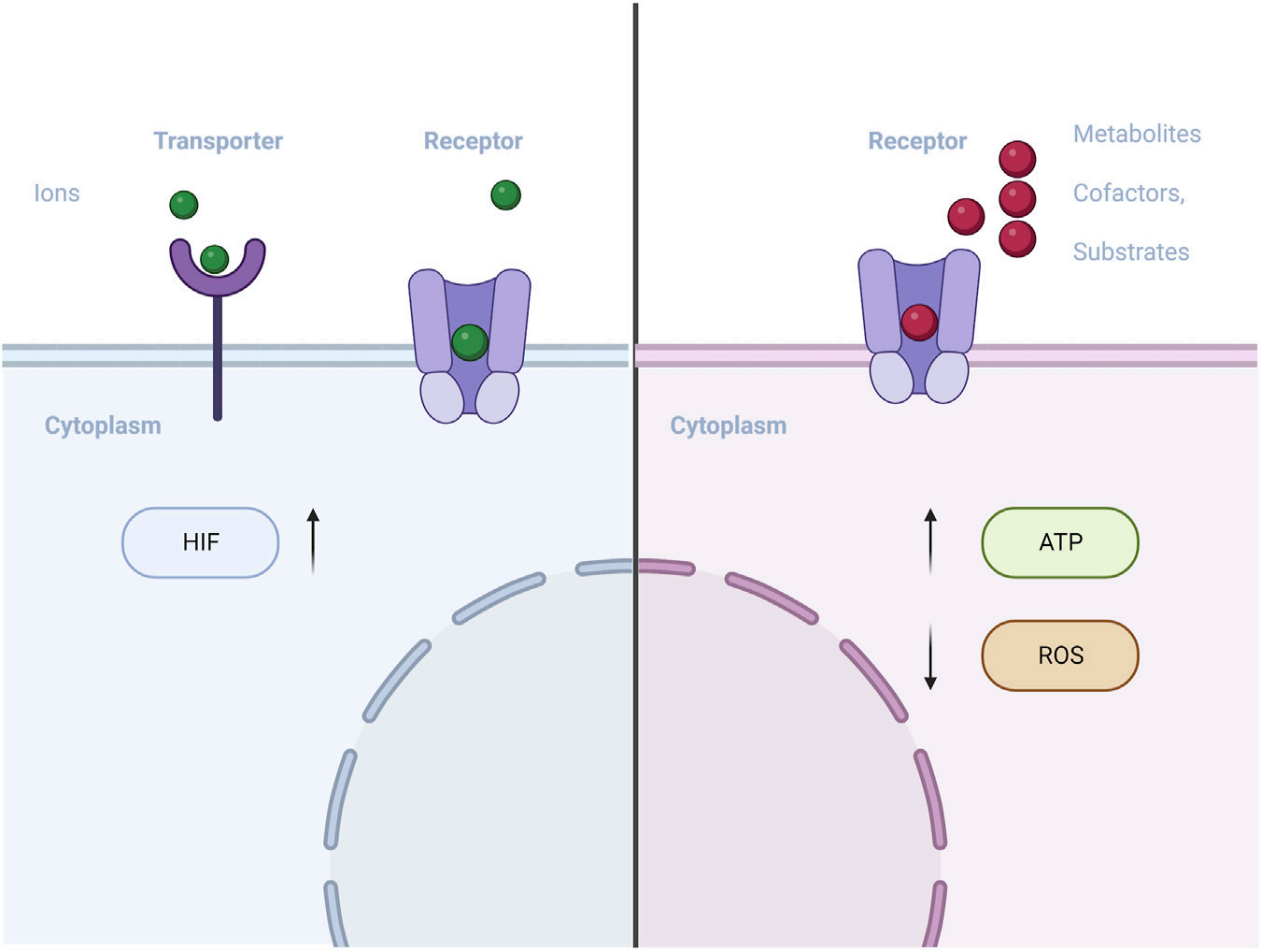
Schematic of materials with metabolic regulators (metal ions/regulatory metabolite) influences intracellular metabolic processes.

An increasing amount of evidence suggests that degradable biomaterials can communicate with cells by releasing degradation products (Murphy et al., 2014). As these products are progressively released into the extracellular environment, they may contain metabolic regulators such as regulatory metabolites, cofactors, and essential biosynthesis substrates (Figure 4). These components can potentially influence intracellular metabolic processes.

**Hyaluronic acid (HA)** is a large, complex molecule that is found throughout the body, particularly in connective tissue, skin, and joint fluid. It plays an important role in cell metabolism by regulating cell proliferation, migration, and differentiation. One way that HA affects cell metabolism is through its interactions with cell surface receptors, such as CD44 and RHAMM (Misra et al., 2015).T hese receptors bind to HA and activate signaling pathways that can affect cell behavior. For example, HA can promote cell migration by activating the RHAMM receptor, which triggers changes in the cytoskeleton and allows cells to move through tissue (Entwistle et al., 1996). HA also plays a role in the extracellular matrix (ECM), the network of proteins and other molecules that surrounds cells. It helps to regulate the hydration and elasticity of the ECM, which in turn affects cell behavior (Karamanos et al., 2021). Furthermore, HA has been shown to affect mitochondrial function, the organelles responsible for generating energy within cells. Studies have suggested that HA can enhance mitochondrial respiration and ATP production, which can promote cell survival and function (Solis et al., 2016). Researchers designed lacunar hyaluronic acid microcarriers (LHAMC) that rapidly form stable hyaluronic acid NHSester under specific conditions. Chondrocytes cultured on LHAMC undergo unique remodeling of the extracellular matrix, inducing hyaline cartilage regeneration and preventing metabolic transition. LHAMC also inhibit the Wnt pathway, preventing chondrocyte dedifferentiation (Ding et al., 2023).

**Alpha-ketoglutarate (AKG)** plays a crucial role in cellular metabolism. While it is an essential molecule in the Krebs cycle that regulates the rate of the citric acid cycle, its functions extend beyond energy production. AKG also acts as a nitrogen scavenger, playing a vital role in nitrogen metabolism. It serves as a precursor to produce glutamate and glutamine, which are critical for protein synthesis. In muscles, AKG has been shown to inhibit protein degradation, contributing to the maintenance of muscle mass. Additionally, AKG has been shown to have antioxidant properties, protecting cells against oxidative stress (Wu et al., 2016; Rhoads and Anderson, 2020). The AKG possesses an extensive scope of applications, Yousaf et al. (Barrett and Yousaf, 2008) Polycondensation of AKG to one of the triols glycerol, 1,2,6 hexanediol, or 1,2,4-butanetriol results in poly (triol-α-ketoglutarate) with extensive mechanical and chemical properties. Furthermore, the hydrolytic degradation of the poly( triol-α-ketoglutarate) series occurred within a range of 2 –8 days in phosphate-buffered saline solutions. The repeat units of the series comprise ketones that can react with diverse oxyamine-terminated molecules to form stable oxime linkages in post-polymerization modifications. In addition to its metabolic functions, AKG has also been shown to exhibit a pro-osteogenic effect on osteoblast cell lines. The degradation products released by AKG can activate the JNK and mTORC1/S6K1/S6 signaling pathways. These signaling pathways play a critical role in regulating osteoblast differentiation and function, ultimately leading to bone formation. The activation of the JNK pathway has been shown to promote osteoblast differentiation and mineralization, while the mTOR/S6K1/S6 pathway regulates protein synthesis, which is essential for bone formation. By activating these keys signaling pathways, AKG degradation products promote osteoblast differentiation and function, leading to the formation of new bone tissue. These findings suggest that AKG may have potential therapeutic applications in bone-related diseases and injuries, promoting bone regeneration and recovery. (Zurek et al., 2019). Finally, AKG has been shown to reduce the overall levels of two critical histone modifications, H3K9me3 and H3K27me3, that are associated with cell senescence, organismal aging, and age-related osteoporosis. AKG has been proposed to have a geroprotective effect through various mechanisms, including being a substrate for DNA and histone demethylases, having direct antioxidant properties, and mimicking the effects of caloric restriction and hormesis. AKG also can stimulate the production of ROS by mitochondria, which may have beneficial effects by inducing defensive mechanisms that improve resistance to stressors and age-related diseases, according to the hormesis hypothesis (Bayliak and Lushchak, 2021).This ability enables AKG to protect and rejuvenate aged MSCs, while also promoting their proliferation vitality, migration *in vitro* and osteogenic differentiation. (Y. Wang et al., 2020).

**Citric acid** is a natural metabolic regulator that plays a key role in the Krebs cycle. It has been identified in the degradation products of citrate-based biomaterials (CBBs) and has been shown to enhance the bioenergetics of MSCs, thus promoting their osteoblast differentiation. To produce energy for MSC differentiation and bone formation, materials that regulate energy metabolism are studied, with citric acid-based materials being a focus due to its importance in TCA metabolism and its role in regulating bone mineralization. Citric acid plays a crucial role in forming a biomineralization network and its secretion is necessary for osteogenic differentiation and mineralization. Exogenous citric acid promotes MSC osteogenic differentiation and mineralization by regulating TCA metabolism to improve energy metabolism. (Ma et al., 2018a). Citrate, an intermediate metabolite, is recognized as a well-known intracellular molecule that plays a significant role in regulating energy homeostasis (Zara et al., 2022). Citric acid plays a vital role in cellular metabolism by regulating the activity of important enzymes involved in both catabolic and anabolic pathways. Additionally, it can be converted to acetyl-CoA, which serves as a direct substrate for the biosynthesis of fatty acids and the acetylation of histones (Williams and O'Neill, 2018; Iacobazzi and Infantino, 2014). Citric acid-based bone repair materials have been developed, exhibiting excellent osteoinductive and osteogenic activities. Ma et al. have designed a citrate/phosphoserine-based photoluminescent biodegradable polymer (BPLP-PSer), which was fabricated into BPLP-PSer/hydroxyapatite composite microparticulate scaffolds that demonstrated significant improvements in bone regeneration (Ma et al., 2018b). Tian et al. developed tannin modified HA (THA) and silver/tannin modified hydroxyapatite (Ag-THA). They then mixed Ag-THA with non-antimicrobial polyurethane (PU) to create a composite material called PU/Ag-THA. This composite material showed promising results for bone regeneration. Guo et al. have developed citrate-based tannin-bridged bone composites, which demonstrate excellent biocompatibility, bone conduction, and mechanical properties. To effectively connect the organic and inorganic phases, They designed a citrate-based biodegradable polymer, the residual functional groups of immobilized TA substituents are covalently bonded to poly( octamethylene citrate) (Guo et al., 2020).U pon degradation, the released citrate enters hMSCs through the plasma membrane transporter SLC13a5, modulating energy-producing pathways by enhancing OXPHOS and inhibiting glycolysis. As a result, intracellular ATP levels are significantly increased, providing support for the high metabolic demand during the osteogenic differentiation of hMSCs. This increase in ATP levels promotes the expression of Runx2 and the production of extracellular matrix related to bone formation.

**FIGURE 5:**
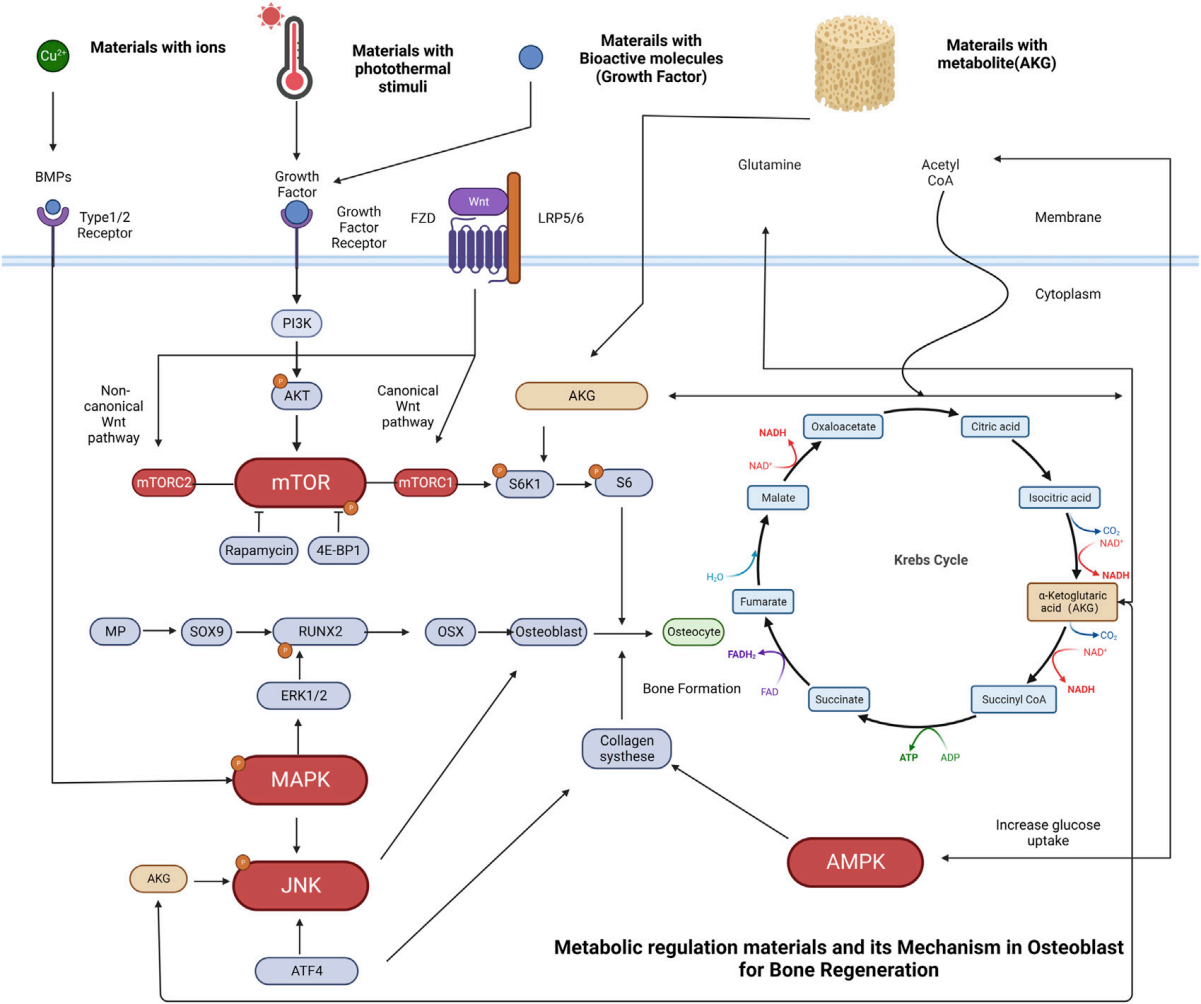
Schematic representation of osteoblasts metabolic regulation and some typical examples of materials related to bone regeneration. **(A)** Cu^2+^ has been incorporated into biomaterials such as bioactive glass, promote osteogenesis by increasing BMP-2 secretion. The binding of BMP-2 ligand to the receptor initiates a signaling cascade that leads to the phosphorylation of the MAPK pathway, with a specific focus on the extracellular signal-regulated kinases (ERK1/2). These pathways have been identified as key regulators of transcriptional events that mediate osteoblast differentiation. Phosphorylation of RUNX2 by ERK1/2 is an important step in this process. **(B)** Materials with photothermal stimuli can regulate growth factors. Upon binding of a growth factor ligand to the receptor, a signaling cascade is initiated, resulting in the phosphorylation of AKT by PI3K and activation of the mTOR pathway. mTORC1 activation leads to the phosphorylation of S6K1, which then phosphorylates the S6 protein, facilitating biosynthetic pathways and protein synthesis that promote cell growth. **(C)** Materials with growth factors can also promote ossification through the mTORC1-dependent control of the S6K1/S6 axis **(D)** Materials with regulatory metabolite [e.g., Alpha-ketoglutarate (AKG)] release degradation products that exert a pro-osteogenic effect in osteoblast cell lines through activation of JNK and mTOR/S6K1/S6 signaling pathways. AKG is a vital molecule in the Krebs cycle that regulates the citric acid cycle rate, functions as a nitrogen scavenger, and supplies glutamate and glutamine for protein synthesis while hindering protein degradation. In addition, AKG plays a crucial role in the Krebs cycle, regulating the rate of the citric acid cycle and enhancing glucose intake. The increasing glucose intake activates AMPK, resulting in the upregulation of collagen type I expression, which is fundamental for bone formation and mineralization.

Nowadays, the treatment of bone defects is a challenging and costly process with unpredictable outcomes. Biodegradable scaffolds have emerged as a promising solution, but their clinical success depends on several factors, including biocompatibility, biodegradability, osteoconductivity, low immunogenicity, and non-infectiousness. While advanced materials have been developed, traditional biodegradable materials such as natural and synthetic polymers, ceramics, and metals still offer major advantages, including natural adhesion ligands, excellent mechanical strength, and good osteoconductivity. Although these materials have some limitations, they continue to serve as the basis for the development of a new generation of degradable materials. The current trend in bone defect repair involves the use of composite materials that integrate the advantages of different materials, such as natural and synthetic polymers, ceramics, and metals (Figure 5). For instance, an AKG-based bioactive polymer has shown potential as a more effective approach to bone regeneration by activating JNK and mTOR/S6K1/S6 signaling pathways in osteoblasts through the degradation products released by the polymer. The development of intelligent materials is expected to gradually resolve these issues. However, transitioning a new type of bone defect repair scaffold from the laboratory to the clinic is a challenging journey that necessitates interdisciplinary collaboration among researchers and scientists. Table 1

**TABLE 1:** Summary of biomaterials involved in the metabolic regulation in bone regeneration.

Strategy for biomaterials involved in the metabolic regulation	Materials examples	Signaling pathways	Ref.
Materials with advanced	CTBCs	MAPK	Guo et al. (2020)
Physicochemical characteristics	DBD-modified Ti plates	AMPK	Zuo et al. (2013)
	Hybrid hydrogel with tunable stiffness	AMPK	Zhou et al. (2023)
**Materials binding exogenous stimuli.**	Near infrared-responsive hydrogels	mTOR	Zhang et al. (2019a)
1) Photothermal stimulation	Temperature-responsive hydrogel	mTOR	Yang et al. (2020b)
2) Electrical stimulation	Electroactive composite scaffold with Locally expressed osteoinductive factor magnetic microcarrier	mTOR	Maharjan et al. (2020)
3)Magnetic stimulation	Scaffolds with SMF	JNK	Go et al. (2020)
		MAPK	Go et al. (2020)
			Yun et al. (2016)
**Materials with metabolic regulators**	CPC/GO-Cu	MAPK	Zhang et al. (2019b); Liang et al. (2021)
1)Metal Ions	Morin-zinc complexes	MAPK	Vimalraj et al. (2019)
2) Bioactive molecules	Porous zinc scaffolds	MAPK	Cockerill et al. (2020)
3) Regulatory Metabolite	BMP-2/Mg2+ codelivery system	MAPK	Lin et al. (2022)
	Scaffolds with BMP-2 and alendronate	MAPK	Li et al. (2015)
	Microcarriers encapsulate the drugs/bioactive factors	JNK/MAPK	Ding et al. (2022)
	BPLP-PSer	MAPK	Tran et al. (2015), Ma et al. (2018a)
	poly( triol α-ketoglutarate)	JNK/mTOR	Barrett and Yousaf, (2008)
**Materials with AI**	Gd-BTO NPs	calcineurin/NFAT	Wang et al. (2020)

citrate-based tannin-bridged bone composites, (**CTBCs**); static magnetic field, (**SMF**); Graphene Oxide-Copper Nanocomposite-Coated Porous CaP Scaffold, (**CPC/GO-Cu**); citrate/phosphoserine-based photoluminescent biodegradable polymer, (**BPLP-PSer**); electroactive biocomposite of poly( lactic-co-glycolic acid) mixed with gadolinium-doped barium titanate nanoparticles, (**Gd-BTO NPs**); Dielectric barrier discharge, (**DBD**).

## 5 Discussion

Currently, materials for bone regeneration have advanced significantly. However, achieving high biocompatibility and optimizing the regulation of cell recruitment, migration, adhesion, proliferation, differentiation, and the precise control of gene expression and metabolic regulators release remain challenging areas that require further research. Additionally, verifying the safety of clinical applications is a critical issue that must be addressed in future studies (S. Wei et al., 2020). Biomaterials involved in the metabolic regulation have emerged as a promising approach to promote bone regeneration. These materials are designed to release metabolic regulators, such as growth factors or signaling molecules, at the site of bone injury or disease to stimulate bone growth and healing. The development of intelligent biomaterials involved in the metabolic regulation that can provide precise and targeted release of these regulators is crucial for their effective use in bone regeneration. In recent years, researchers have made significant progress in this area, with the development of new materials with advanced physicochemical characteristics, exogenous stimuli or metabolic regulators to trigger the bone regeneration. These advancements in the field of biomaterials involved in the metabolic regulation offer great potential for the development of safe and effective bone regeneration therapies that can be tailored to the specific needs of individual patients. It is important for researchers from various fields to continue collaborating to advance the development of these materials and bring them from the laboratory to clinical application. Artificial intelligence (AI) is a rapidly developing field that has the potential to revolutionize many aspects of our lives. AI can be used to design and develop intelligent materials for regulating metabolism in osteoblasts, which are the cells responsible for bone formation. AI can be used to analyze large datasets of information on the properties and behaviors of different materials, enabling researchers to identify which materials are most effective for promoting bone regeneration. Designing intelligent biomaterials involved in the metabolic regulation that are superior to autologous bone and allograft bone requires advanced material science, biomedicine, and consideration of clinical needs. Such materials should aim to achieve the following objectives: (1) meet requirements for biocompatibility, mechanical stability, biodegradability, osteoinductivity, osteoconductivity, osseointegration, and osteogenic ability; (2) possess physical and chemical properties that enable efficient bone regeneration, including a porous structure and hierarchical gradient structure similar to bone tissue; (3) specifically release metabolic regulators, accurately simulating the bone regeneration process; and (4) The integration of real-time imaging technology and artificial intelligence with the design and preparation of advanced materials has the potential to develop personalized treatment plans for diverse clinical needs.

However, achieving the aforementioned goals requires further interdisciplinary collaboration among researchers from various fields to promote the advancement of bone regeneration materials. We anticipate that the development of intelligent materials and materials designed for the precise release of metabolic regulators will help address these challenges. Furthermore, the transition from laboratory to clinic for new bone defect repair scaffolds is a long and challenging journey that necessitates the collective efforts of scientists and researchers from numerous fields. Bone regeneration is a critical area of research with significant implications for patient care. The development of effective bone regeneration materials can provide hope for individuals who have suffered from bone injuries or diseases, such as osteoporosis, osteomyelitis, or bone tumors. However, the path from laboratory discovery to clinical application is long and challenging. Thus, this review seeks to provide guidance and inspiration for researchers working on bone regeneration materials by discussing the latest progress in the field, highlighting key challenges, and proposing potential solutions. By doing so, this review aims to support the development of safe and effective bone regeneration materials, which have the potential to improve the quality of life for many patients in need.
